# Chlorpheniramine-induced anaphylaxis: Two case reports and a retrospective review of pharmacovigilance database

**DOI:** 10.1097/MD.0000000000018369

**Published:** 2019-12-16

**Authors:** So-Hee Lee, Youngsoo Lee, Seong-Dae Woo, Ko-Eun Doo, Chae-Yeon Ha, Young-Hee Lee, Young-Min Ye

**Affiliations:** a Department of Allergy and Clinical Immunology; b Regional Pharmacovigilance Center, Ajou University Hospital, Suwon, Korea.

**Keywords:** adverse drug reactions, anaphylaxis, antihistamines

## Abstract

**Rationale:**

Anaphylaxis is a serious allergic reaction which could be life-threatening. To date, it could be diagnosed by causality between clinical manifestations and triggers. But it is not always easy to find out the clue. Chlorpheniramine maleate (peniramin) is known to safe and it is an antihistamine commonly used to treat almost the whole allergic disease, including urticaria and allergic rhinitis. We recently experienced 2 cases of chlorpheniramine induced anaphylaxis. To document suspected cases of chlorpheniramine-induced adverse reactions, we analyzed a database spontaneously reported adverse drug reactions in the Ajou Regional Pharmacovigilance Center from 2011 to 2017.

**Patient concerns:**

Two female patients presented urticaria and abdominal pain right after chlorpheniramine injection.

**Diagnoses:**

Both patients were diagnosed with symptoms. One patient confirmed by assistance with tryptase level and another one confirmed cross-reactivity by skin tests.

**Interventions:**

One patient was instructed to avoid future administration of chlorpheniramine. The other patient was advised not to take chlorpheniramine, and piperazine derivatives including cetirizine/levocetirizine, but piperidine derivatives such as fexofenadine, loratadine, and ebastine can be available.

**Outcomes:**

The patients fully recovered after prompt treatment for anaphylaxis. After that, no recurrences were observed at the following. Among 54 patients with chlorpheniramine-induced adverse drug reactions from the Pharmacovigilance Center database, 17 (31.5%) were reported as anaphylaxis.

**Lessons:**

Physicians should be aware chlorpheniramine could be a cause for allergic reaction. In addition, we suggest that serum tryptase level, skin prick test, and intradermal test could be considered as a supplementary test for diagnosing chlorpheniramine anaphylaxis and cross-reactivity should also be considered.

## 1 Introduction

Chlorpheniramine is one of the most classical H1-antihistamines (AHs) and is commonly used for various allergic reactions. AHs are usually well-tolerated, although several side effects which were classified in type A reaction of adverse drug reactions (ADRs), such as drowsiness, mouth dryness, edema, and changes in appetite/weight are well recognized.^[1]^ Thus, we often overlook the hypersensitivity reactions caused by AHs. However, rarely, urticaria caused by different AHs has been reported.^[2–4]^

Anaphylaxis is an important clinical condition that may lead to death, and medication including antibiotics, nonsteroidal anti-inflammatory drugs, and radiocontrast media is the major cause of anaphylaxis in adults^[5]^ Diagnosis of anaphylaxis is always easy, because it is usually based on clinical symptoms developed after the exposure to possible triggers. Cutaneous manifestations, such as acute urticaria, angioedema, and itchy erythema, are noted in almost patients with anaphylaxis. Because chlorpheniramine is the only AHs available for injection, to control acute urticaria, physicians are used to prescribing chlorpheniramine injection for the patients with urticaria and/or anaphylaxis. To date, immediate hypersensitivity reactions caused by chlorpheniramine have been rarely reported.^[6,7]^ Particularly, there has been only a few case reports on anaphylaxis induced by chlorpheniramine.^[8]^

Here, we report 1 case of chlorpheniramine-induced anaphylaxis which diagnosis has been supported by elevated serum tryptase and another case of that which diagnosed with cross-reactivity. We reviewed ADRs associated with chlorpheniramine administration from the database of the Ajou Regional Pharmacovigilance Center from 2011 to 2017.

## 2 Case presentation

### 2.1 Case 1

A 54-year-old female was referred to the emergency department due to skin rash appeared after taking rabeprazole, clarithromycin, and amoxicillin orally to treat *Helicobacter pylori* infection. Upon being administered 4 mg of intravenous chlorpheniramine maleate (Peniramin, Yuhan Pharma Co, Ltd, Seoul, Korea), she immediately experienced urticaria, abdominal cramping, nausea, and diarrhea. The patient had no other histories of medical or allergy. Her vital signs were normal, and blood tests revealed leukocyte count of 11,000 × 10^3^ cells/μL (neutrophils, 59.4%; lymphocyte, 35.5%; monocyte, 4.5%; eosinophils, 0.4%) and other blood cell counts within normal ranges. After receiving emergency care for anaphylaxis, including fluid resuscitation and intravenous steroids treatment with dexamethasone (Dexa-S, Ilsung Pharma Co, Ltd, Seoul, Korea), she was fully recovered. In a laboratory test, serum total immunoglobulin E (IgE) levels had not increased (33 KU/L), while serum tryptase levels had increased to 16.8 mg/L (0.00–11.40 mg/L). A test for serum-specific IgE to amoxicillin was negative. On the basis of the clinical manifestations that developed immediately after exposure to chlorpheniramine and elevated serum tryptase levels, we diagnosed the patient as having experienced chlorpheniramine-induced anaphylaxis. In final follow up after 3 months of this episode, she had no problem and continued to avoid chlorpheniramine.

### 2.2 Case 2

A 50-year-old female was referred to the emergency department due to acute exacerbation of chronic spontaneous urticaria. After receiving 4 mg of intravenous chlorpheniramine maleate (Peniramin, Yuhan Pharma Co, Ltd), the patient experienced an aggravation of urticaria and abdominal pain. After receiving emergency care for anaphylaxis, including fluid resuscitation and intravenous steroids treatment with dexamethasone (Dexa-S, Ilsung Pharma Co), she was fully recovered. Previously, she had experienced an inject-site rash after administration of intramuscular chlorpheniramine and fully recovered after administration of intravenous dexamethasone (Dexa-S, Ilsung Pharma Co, Ltd), although she had no problems with the drug when administered orally. She had no other medical histories. Her blood pressure was 142/81 mm Hg, and blood analysis revealed leukocyte count of 7400 × 10^3^ cells/μL (neutrophils, 50.1%; lymphocyte, 41.1%; monocyte, 8.1%; eosinophils, 0.4%) and other blood cell counts within normal ranges. Serum total IgE levels had not increased (63 KU/L), and the results of specific IgE to common inhalant allergens were all negative. To identify the culprit drug and a safe alternative drug for treatment of her chronic spontaneous urticaria, we performed skin tests with AHs, including chlorpheniramine (Peniramin), fexofenadine (Allegra, Teva-Handok Pharma Co, Ltd, Seoul, Korea), cetirizine (Zyrtec, Korea UCB Co, Ltd, Seoul, Korea), levocetirizine (Xyzal, Korea UCB Co, Ltd), loratadine (Clarityne, Bayer Korea Ltd, Seoul, Korea), and ebastine (Ebastel, Boryung Pharma Co, Ltd, Seoul, Korea). Intradermal tests (IDTs) with chlorpheniramine, levocetirizine, and cetirizine showed positive responses at a 1:10 dilution (4-mm × 4-mm wheals) and therapeutic doses (4-mm × 4-mm and 5-mm × 5-mm wheals), respectively. Oral provocation test with levocetirizine was positive (Table 1). Taken together with her clinical history and results from skin tests and oral provocation tests, we diagnosed the patient as having experienced chlorpheniramine-induced anaphylaxis and having cross-reactivity to cetirizine/levocetirizine (piperazine derivatives). Since then, the patient no longer experiences anaphylaxis and her urticaria has been well controlled with fexofenatine treatment.

**Table 1:**
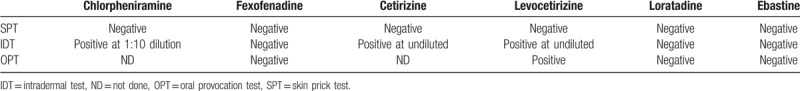
The results of skin tests and oral provocation tests in the second case.

## 3 Chlorpheniramine-induced ADRs in a Regional Pharmacovigilance Center database in Korea

**Figure 1:**
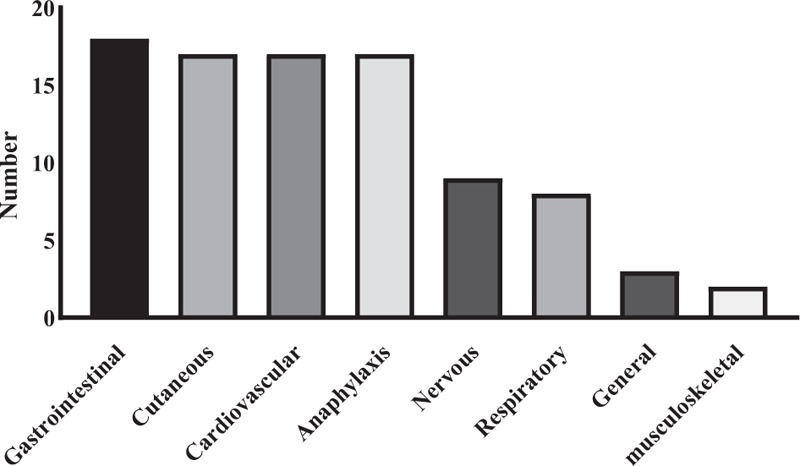
Clinical manifestations of patients experiencing adverse drug reactions to chlorpheniramine.

Chlorpheniramine-associated ADRs were collected from the Ajou Pharmacovigilance Center database for the period from January 2011 to April 2017. Table 2 lists the clinical characteristics of patients with ADRs to chlorpheniramine. In total, 91 type B ADRs in 54 patients were classified as having at least possible causality based on the World Health Organization-Uppsala Monitoring Center causality categories. Figure 1 depicts the clinical manifestations exhibited by patients with chlorpheniramine-induced ADRs. Among the 54 patients, 17 (31.5%) manifested anaphylaxis distinct from the current 2 cases. Among these 17 individuals, 88.2% were exposed to chlorpheniramine via intravenous injection, and 11.8% were treated by intramuscular injection. No anaphylaxis cases were reported for oral administration of chlorpheniramine. Shock (64.7%) was the most common manifestation, followed by dyspnea (29.4%), nausea (23.5%), abdominal pain (17.7%), palpitation (11.8%), angioedema (11.8%), urticaria (11.8%), chest discomfort (5.9%), headache (5.9%), and vomiting (5.9%), as anaphylactic symptoms, in that order. Among the 54 patients, only 5 underwent skin prick tests (SPTs)/IDTs with AHs, including culprit drugs: 1 patient showed a positive response in SPT, and 4 patients showed positive response in IDTs. Serum tryptase level had been checked in 3 patients, and 1 of them had increased tryptase level.

**Table 2:**
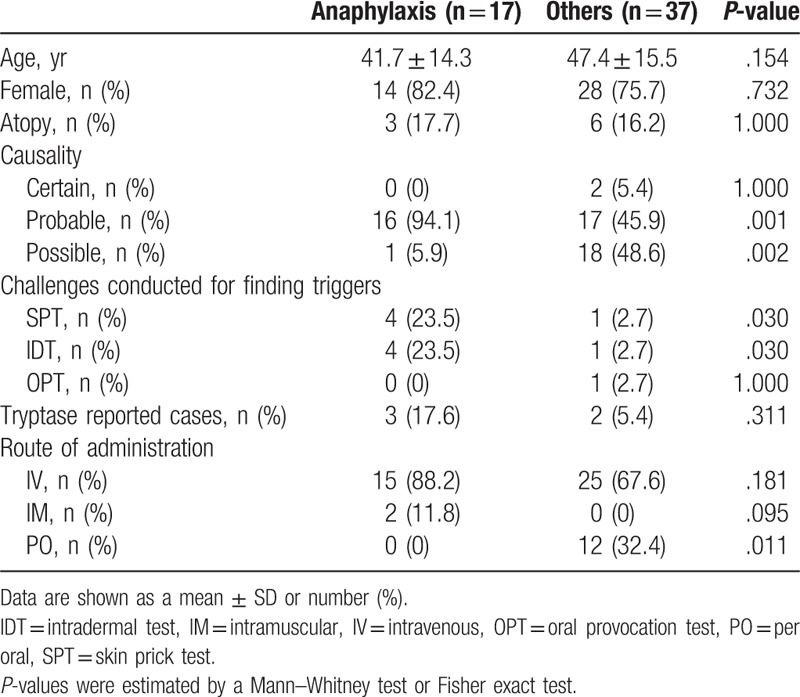
Clinical characteristics of chlorpheniramine-induced anaphylaxis in comparison to chlorpheniramine-induced adverse drug reactions other than anaphylaxis at the Ajou Pharmacovigilance Center.

## 4 Discussion

We report 2 cases of chlorpheniramine-induced anaphylaxis diagnosed by clinical manifestations and increased serum tryptase level or a positive reaction on IDT. Furthermore, we reviewed the records of 17 patients who experienced chlorpheniramine-induced anaphylaxis at the Ajou Pharmacovigilance over the past 6 years. These findings indicated that chlorpheniramine is no longer an ignorable culprit of drug-induced anaphylaxis.

Recently, a case of anaphylaxis caused by intravenous administration of chlorpheniramine confirmed by IDT and basophil activation test was reported in Korea.^[8]^ Similarly, we identified chlorpheniramine as the confirmative cause of anaphylaxis in the 2 patients in this report according to an increase in serum tryptase level and positive responses in IDTs for chlorpheniramine. Although the precise mechanisms of chlorpheniramine allergy are unknown, based on the results of IDT and basophil activation tests and of elevated tryptase levels, an IgE-mediated reaction or direct basophil or mast cell activation may be involved.

The diagnosis of anaphylaxis is based on clinical symptoms that develop when an individual is exposed to a cause, and it is not easy to confirm. SPT/IDT and oral provocation tests are well known as useful tests for identifying culprits of immediate hypersensitivity reactions. However, there is a risk-benefit concern to these tests due to the possibility of recurrence. Meanwhile, serum tryptase level has been found to be an alternative and supplementary marker for diagnosing severe anaphylaxis.^[9–11]^ Mature tryptase level generally reflects the magnitude of mast cell activation, as they are elevated during systemic anaphylactic reactions relative to mild anaphylaxis. Thus, an increase in serum tryptase level could be a supplementary diagnostic tool for discriminating severe anaphylaxis with shock.

AHs are classified by their chemical structures into alkylamines, piperazines, piperidines, ethanolamines, ethylenediamines, and phenothiazines.^[12]^ Cross-reactivity between AHs has not been thoroughly evaluated. Some cases of hypersensitivity to multiple AHs have been reported; however, most of them had no structural similarity.^[3,13,14]^ Cross-reactivity in association with hypersensitivity may be explainable and predictable according to the chemical structure of some drugs, although this may not be the case for AHs.^[15]^ Among the drugs that were applied in SPTs/IDTs in the second case described here, chlorpheniramine is an alkylamine derivative; fexofenadine, loratadine, and ebastine are piperidine derivatives; and cetirizine/levocetirizine are piperazine derivatives. As positive responses to the piperazine derivatives in IDTs were observed, we suspect that cross-reactivity due to the structural similarity of AH derivatives may have been involved.

In a review of Pharmacovigilance data, we discovered that most of the patients with chlorpheniramine-induced anaphylaxis had been administered the drug through intravenous injection, whereas other ADRs, such as urticaria/rashes, were generally reported in patients taking the drug orally. According to the literature, the onset time of oral chlorpheniramine is very short at 15 to 30 minutes.^[16]^ Thus, physicians can consider per oral agents of chlorpheniramine rather than intravenous administration, because orally administered chlorpheniramine poses less risk and a similarly quick effect, compared with intravenous injection.

In conclusion, physicians should be aware that chlorpheniramine can cause allergic reactions. We propose that serum tryptase level and SPT/IDT can be of use as supplementary tests for diagnosing chlorpheniramine anaphylaxis and that cross-reactivity between AHs may be involved in adverse reactions, thus warranting consideration from physicians.

## Acknowledgment

The authors thank the patients for their participation in this study.

## Author contributions

**Conceptualization:** Young-Min Ye.

**Investigation:** So-Hee Lee, Young-Min Ye, Youngsoo Lee, Seong-Dae Woo, Ko-Eun Doo, Chae-Yeon Ha, Young-Hee Lee.

**Writing – original draft:** So-Hee Lee.

**Writing – review and editing:** Young-Min Ye.

Young-Min Ye orcid: 0000-0002-7517-1715.
